# Supplementary material for: Glycosylation and stabilization of programmed death ligand-1 suppresses T-cell activity
Source: Nat Commun. 2016 Aug 30;7:12632. doi: 10.1038/ncomms12632 (PMC5013604; doi:10.1038/ncomms12632)
Supplement: Supplementary Information — Supplementary Figures 1-10 and Supplementary Table 1 [file ncomms12632-s1.pdf]

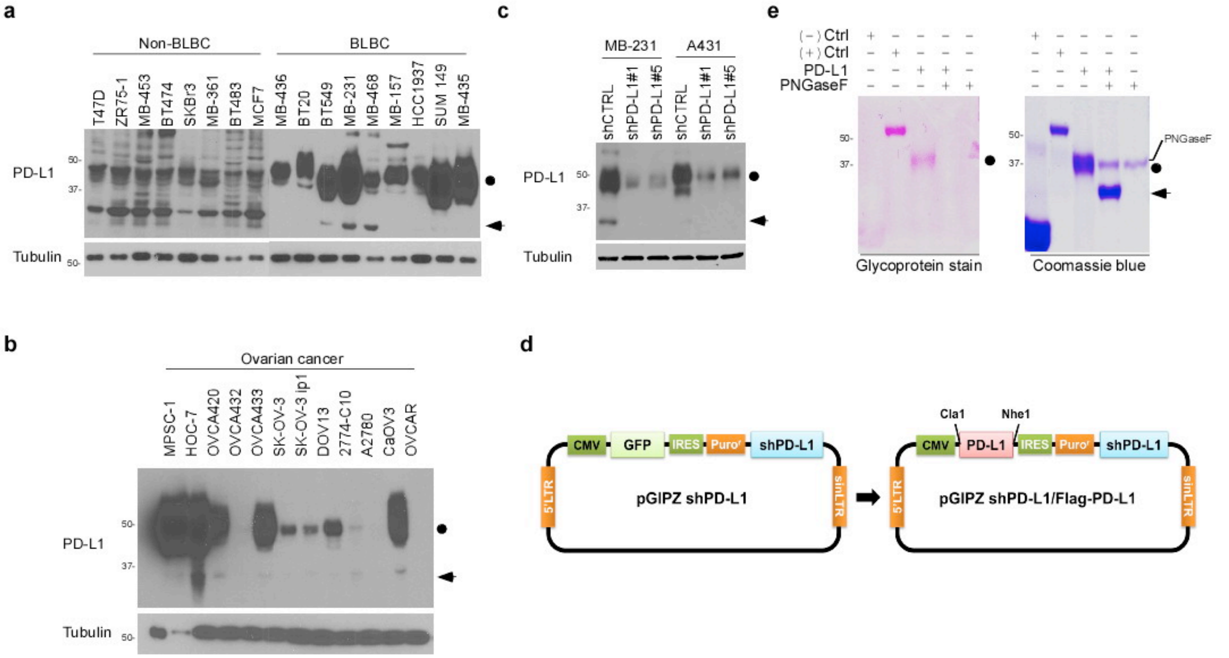

Supplementary Figure 1. PD-L1 is glycosylated in cancer cells. (a) Western blot analysis of PD-L1 in breast cancer cells. **(b)** Western blot analysis of PD-L1 in ovarian cancer cells. **(c)** Western blot analysis of PD-L1 in shCTRL and two independent shPD-L1 stable clones of MDA-MB-231 and A431 cells. shCTRL, control shRNA. **(d)** Dual-expression construct for Flag-PD-L1 and shRNA of PD-L1. **(e)** Glycoprotein staining of purified PD-L1 protein with or without PNGase F treatment. Coomassie blue staining panel represents total amount of PD-L1 protein. The upper bands appear in lane 4 and 5 are from the loading of PNGase F. (–) Ctrl, a control for non-glycoprotein; (+) Ctrl, a control for glycoprotein.

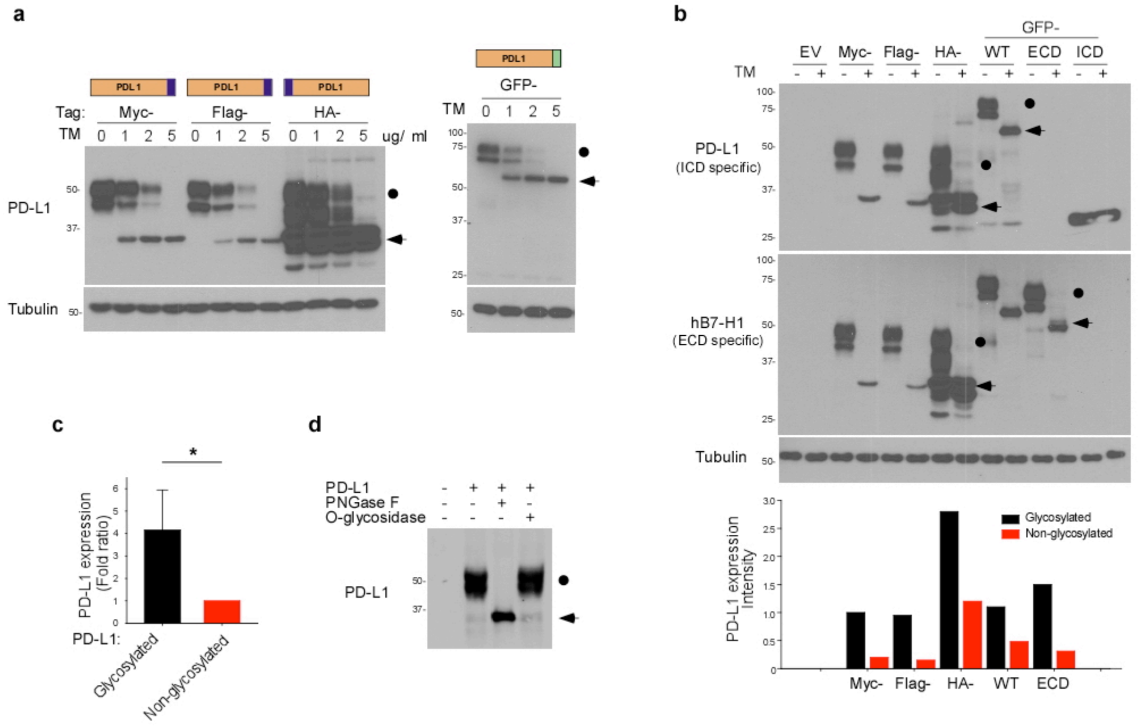

Supplementary Figure 2. Expression of glycosylated and non-glycosylated PD-L1 protein. (a) Western blot analysis of PD-L1-Myc, PD-L1-Flag, HA-PD-L1 and PD-L1-GFP proteins in Tunicamycin (TM) treated cells. **(b)** Western blot analysis of PD-L1-Myc, PD-L1-Flag, HA-PD-L1, PD-L1-GFP WT, ECD, and ICD proteins in TM treated cells. **(c)** The mean of the intensity of glycosylated (black bar) or non-glycosylated PD-L1 (red bar) protein obtained from the bottom of **(b)**. **(d)** Glycosylation pattern of PD-L1 protein in PD-L1 expressing cells. Cell lysates were treated with PNGase F or O-glycosidase and analyzed by Western blot. ECD, extracellular domain; ICD, intracellular domain; Black circle, glycosylated PD-L1; arrowhead, non-glycosylated PD-L1; *P < 0.05 by Student's *t* test. All error bars are expressed as mean ±SD of 3 independent experiments.

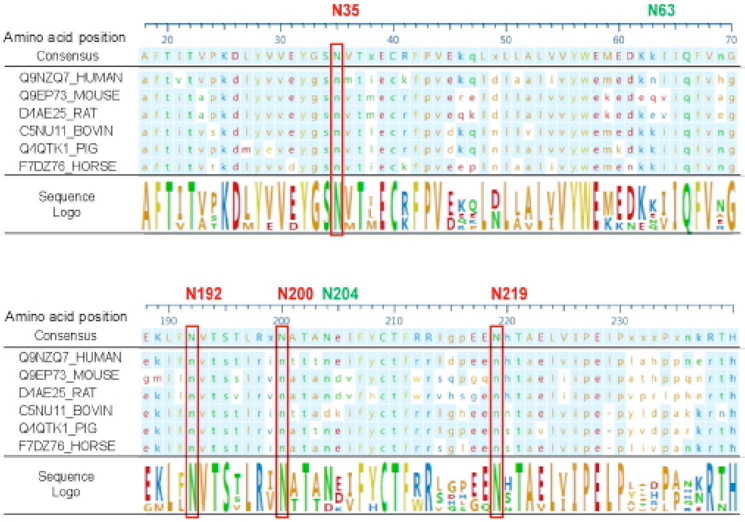

Supplementary Figure 3. N-glycosylation sites of PD-L1 protein. Sequence alignment of the PD-L1 amino acid sequences from different species. Four NXT motifs, N35, N192, N200, and N219 are highlighted in red and two non-NXT motifs, N63 and N204, in green. Red box, conserved NXT motif.

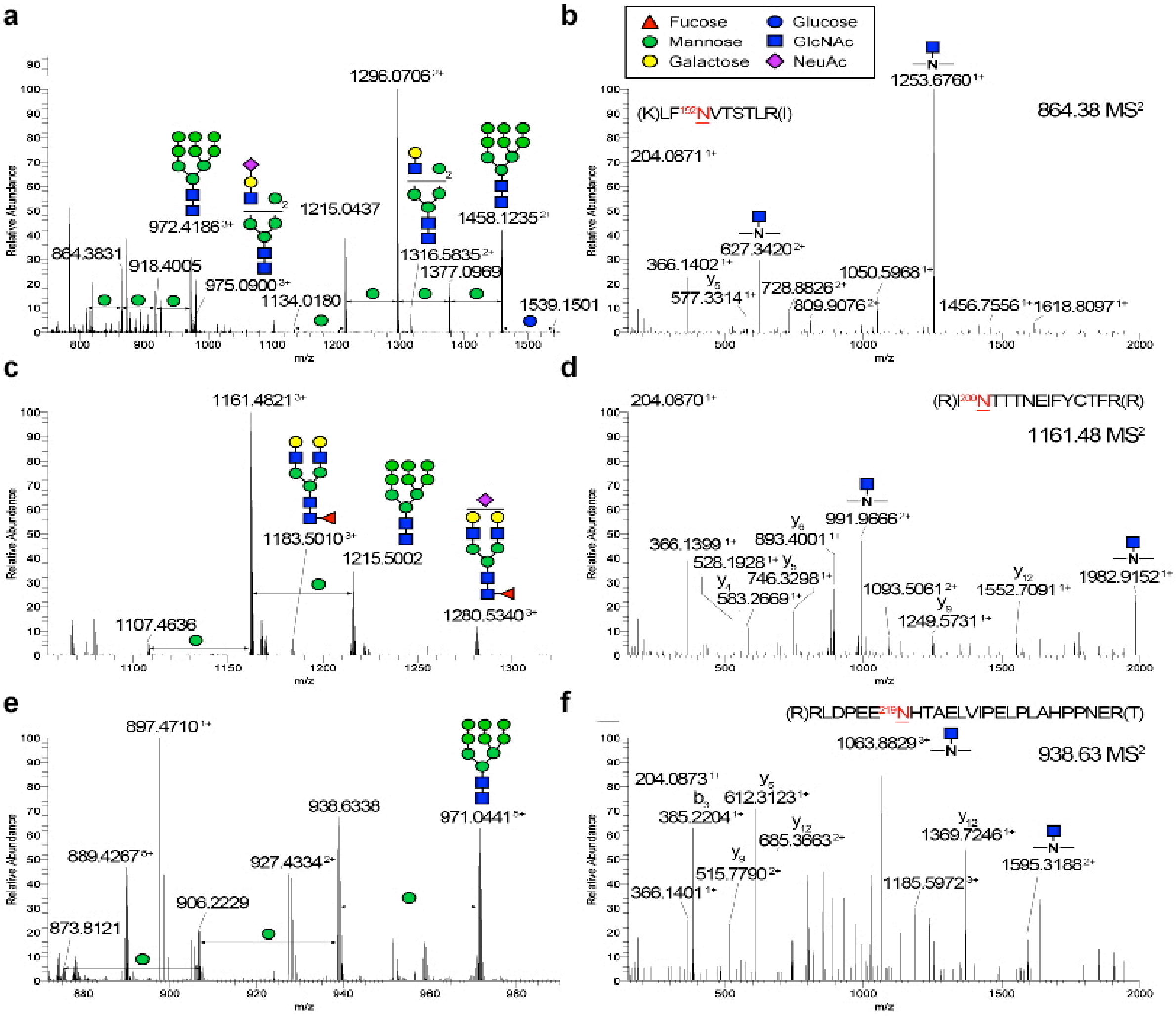

Supplementary Figure 4. N-glycosylation sites of PD-L1 protein. (a)-(f) LC-MS/MS-based identification of *N*-glycopeptides corresponding to each of the four *N*-glycosylation sites, N35, N192, N200, and N219 of PD-L1 (from BT549 cells). The LC-MS profiles **(a, c, and e)** are shown as spectra averaged over a period of elution time (as labeled in figures) when a representative subset of glycoforms were detected. For each *N*-glycosylation site, one representive HCD MS^2 spectrum **(b, d, and f)** is shown to exemplify its identification based on detection of y1 ion (tryptic peptide backbone carrying the GlcNAc attached to the N-glycosylated Asn), along with the b and y ions defining its peptide sequence. The cartoon symbols used for the glycans (see inset) conform to the standard representation recommended by the Consortium for Functional Glycomics: Additional Hex and HexNAc were tentatively assigned as either lacNAc (Gal-GlcNAc) or lacdiNAc (GalNAc-GlcNAc) extension from the trimannosyl core (Man_3-$GlcNAc_2$), which can be core fucosylated.

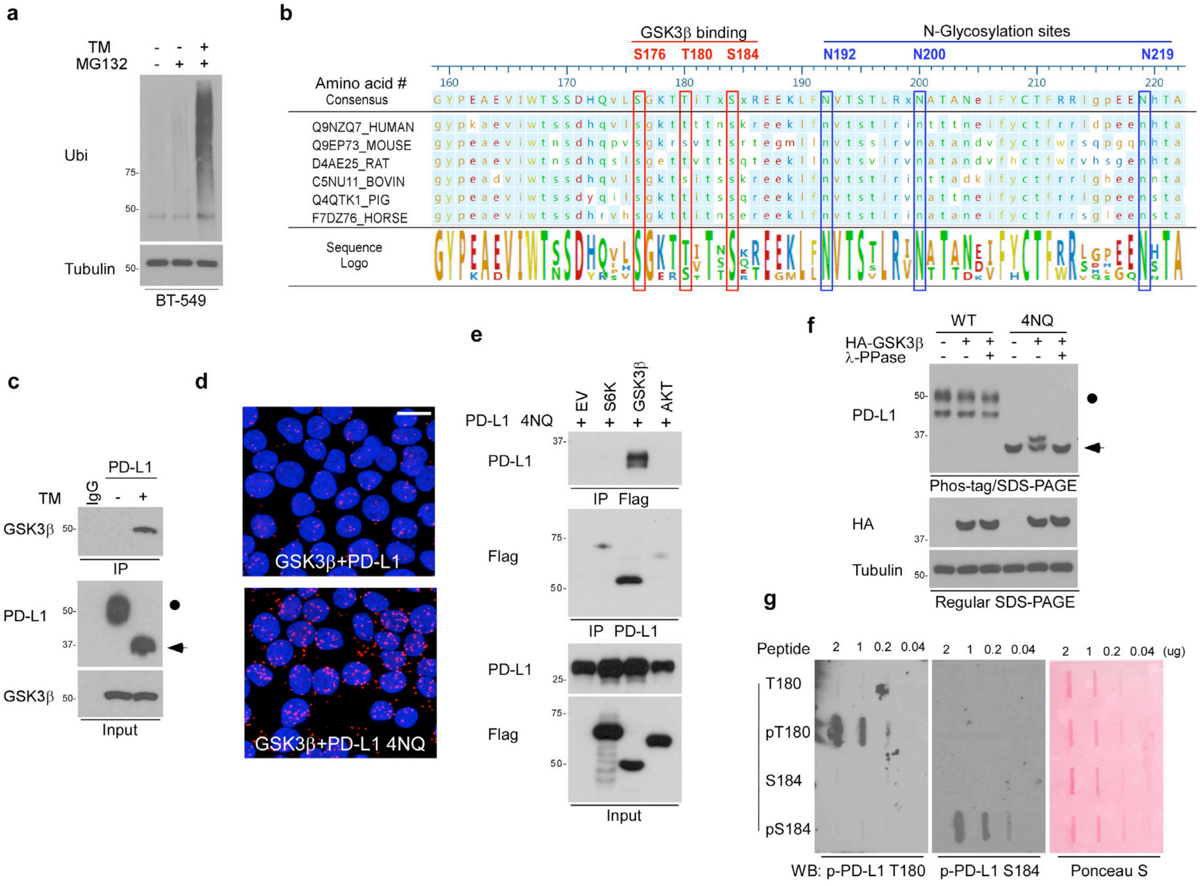

Supplementary Figure 5. GSK3β interacts and phosphorylates PD-L1 (a) Ubiquitination of non-glycosylated PD-L1. Endogenous PD-L1 was immunoprecipitated and subjected to Western blotting with ubiquitin antibody. **(b)** Sequence alignment of the PD-L1 amino acid sequences from different species. Three NXT motifs, N192, N200, and N219 are highlighted in blue and GSK3β phosphorylation motif, S176, T180 and S184, in red. **(c)** Co-immunoprecipitation measuring the interaction of GSK3β and PD-L1. MDA-MB-231 cells were pretreated with 5 μg/ml Tunicamycin (TM) or DMSO for overnight. Endogenous PD-L1 was immunoprecipitated with PD-L1 antibody and subjected to Western blotting with GSK3β antibody. **(d)** PD-L1 WT or 4NQ expressing BT549 cells were immunostained with GSK3β and PD-L1 antibodies and assessed using Duolink® II assay. Red foci indicate interactions between GSK3β and PD-L1 WT or 4NQ proteins. Scale bar, 20 μm. **(e)** The binding affinity between PD-L1 4NQ and GSK3β. HEK 293 cells were transient transfected with indicated plasmid and then subject to immunoprecipitation followed by Western blotting. **(f)** Analysis of PD-L1 phosphorylation using Phos-tag/SDS–polyacrylamide gel (PAGE). HEK 293T cells transfected with PD-L1 WT or PD-L1 4NQ together with HA-GSK3β CA were separated by Phos-tag/SDS–PAGE. Undenatured samples were subject to λ-PPase treatment for 1 hr before resolving on the Phos-tag/SDS–PAGE. **(g)** Characterization of phospho-PD-L1 antibodies. Black circle, glycosylated PD-L1; arrowhead, non-glycosylated PD-L1.

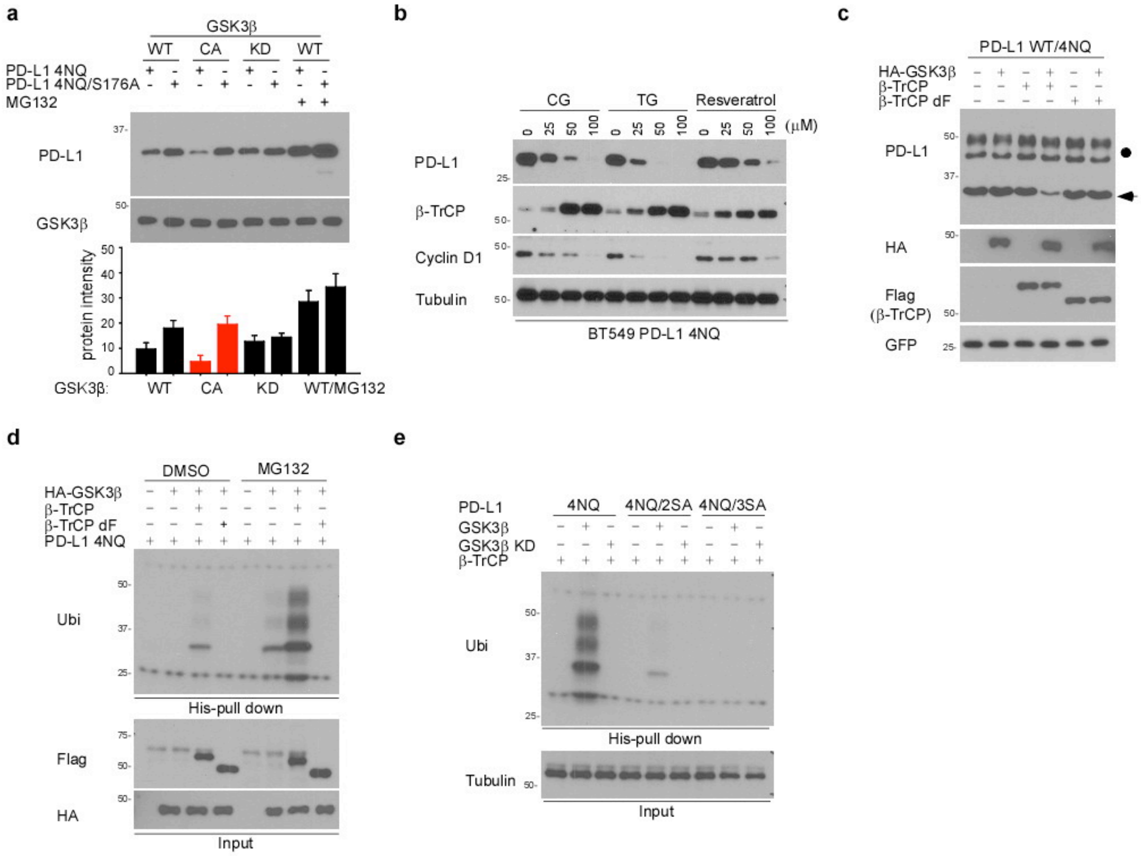

Supplementary Figure 6. GSK3β induces β-TrCP-mediated PD-L1 ubiquitination and degradation. (a) Western blot analysis of PD-L1 4NQ expression. Various GSK3β were transfected together with PD-L1 4NQ to test their ability degrading PD-L1 4NQ. **(b)** BT549 PD-L1 4NQ cells were treated with CG, TG and resveratrol with the indicated concentration for 48 h. Protein expression was analyzed by Western blotting. **(c)** BT-549-PD-L1 4NQ cells were transfected with plasmids expressing HA-GSK3β, Flag-β-TrCP or Flag-β-TrCP dF and analyzed by Western blot. **(d, e)** Ubiquitination of PD-L1 requires GSK3β phosphorylation. GSK3β, PD-L1 4NQ and β-TrCP were transient transfected in HEK 293T cells. Ubiquitinated PD-L1 was pull down by His-tagged Ubiquitin and analyzed by Western blot. Black circle, glycosylated PD-L1; arrowhead, non-glycosylated PD-L1.

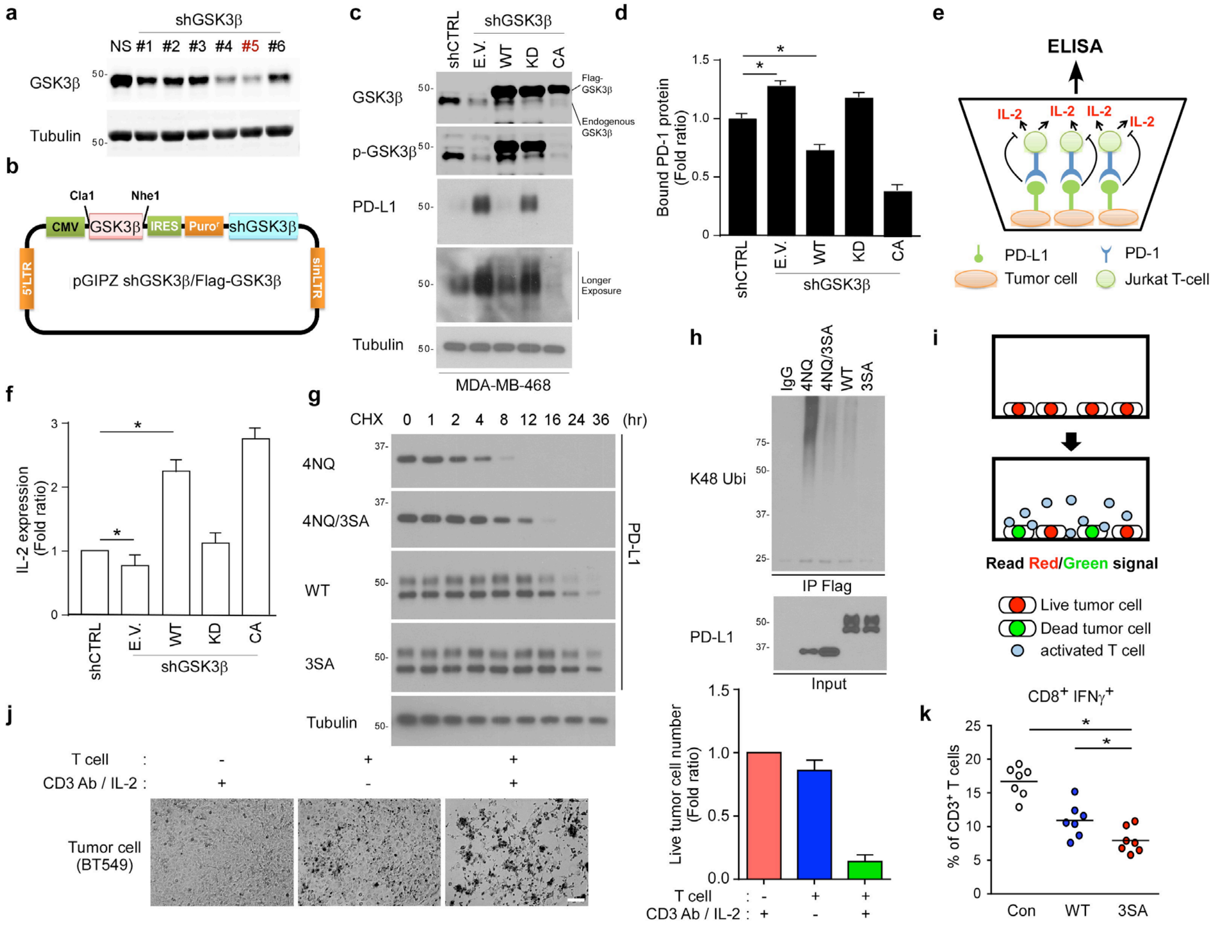

Supplementary Figure 7. (a) Characterization of GSK3β shRNA clones. **(b)** Vector design of GSK3b knockdown and reconstitution. **(c)** Western blot analysis of stable clones with GSK3β variants. **(d)** PD-1 binding assay of GSK3β stable clones from **(c)**. **(e)** Schematic diagram of IL-2 ELISA. PD-L1 stable clones were co-cultured with PD-1 overexpressed Jurkat T cells. IL-2 expression from Jurkat T cells was measured by ELISA. **(f)** Jurkat T cells IL2 expression of GSK3β stable clones from **(c)**. **(g, h)** Western blot analysis of PD-L1 4NQ, 4NQ/3SA, WT, and 3SA protein stability. Cells were treated with or without 20 mM CHX as indicated intervals shown in **(g)**. Protein ubiquitination is shown in **(h). (i)** Schematic diagram of T cell-mediated tumor cell killing assay. Nuclear restricted RFP expressing tumor cells and activated T cell are co-cultured in presence of Caspase 3/7 substrate. T cells were activated with anti-CD3 antibody (100 ng/ml) and IL-2 (10 ng/ml). At 96 hr, RFP and green fluorescent (NucView^{TM} 488 Caspase 3/7 substrate) signal were measured. Green fluorescent cell was counted as dead cell. **(j)** Representative phase images (10x) of BT549 cells and/or activated T cells co-cultures at 96 hr. T cells were activated with anti-CD3 antibody (100 ng/ml) and IL-2 (10 ng/ml). The quantitative ratio of live cells is shown in bar graph (right). Scale bar, 100 μm. **(k)** Intracellular cytokine stain of IFNγ in $CD8^+$ $CD3^+$ T cell populations from the isolated TIL. Con, vector control expressing cells; WT, PD-L1 WT expressing cells; 3SA, PD-L1 3SA expressing cells. * $P < 0.05$ by Student's *t* test. All error bars are expressed as mean ±SD of 3 independent experiments.

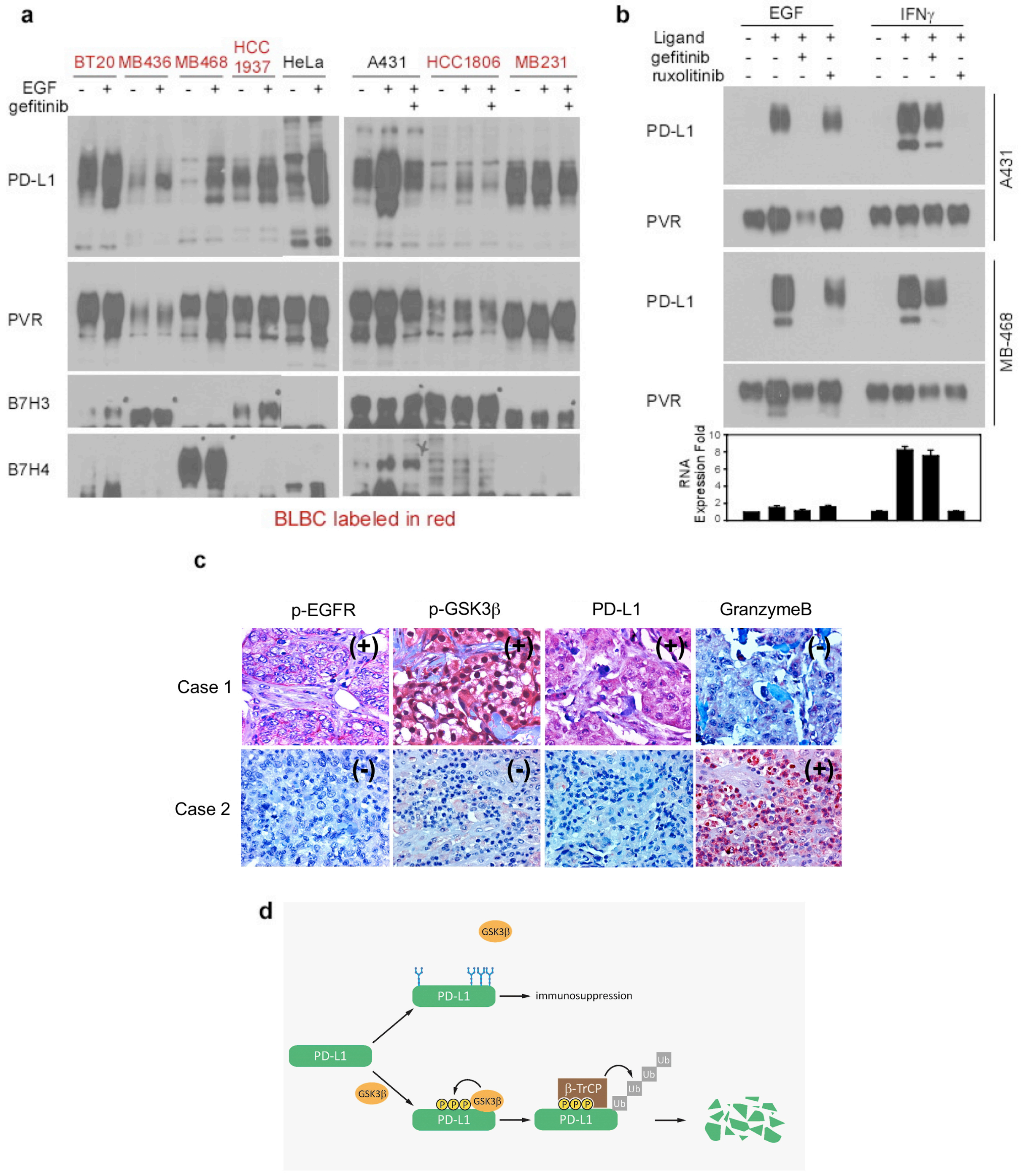

Supplementary Figure 8. EGF induces PD-L1 glycosylation. **(a)** Western blot analysis of PD-L1 glycosylation in various cells. Cells were serum starvation for overnight and then treated with 25 ng of EGF. **(b)** Western blot analysis of PD-L1 expression upon EGF or IFNγ treatment. The mRNA expression of individual treatment was shown in the bottom. **(c)** Representative images from IHC staining of p-EGFR (phosphorylation of Tyr 1068), p-GSK3β (phosphorylation of Ser

9), PD-L1, and granzyme B in primary breast cancer patients. **(d)** A proposed model of EGF mediated PD-L1 stabilization contributing to T cell immune escape. Black circle, glycosylated PD-L1; arrowhead, non-glycosylated PD-L1. All error bars are expressed as mean ±SD of 3 independent experiments.

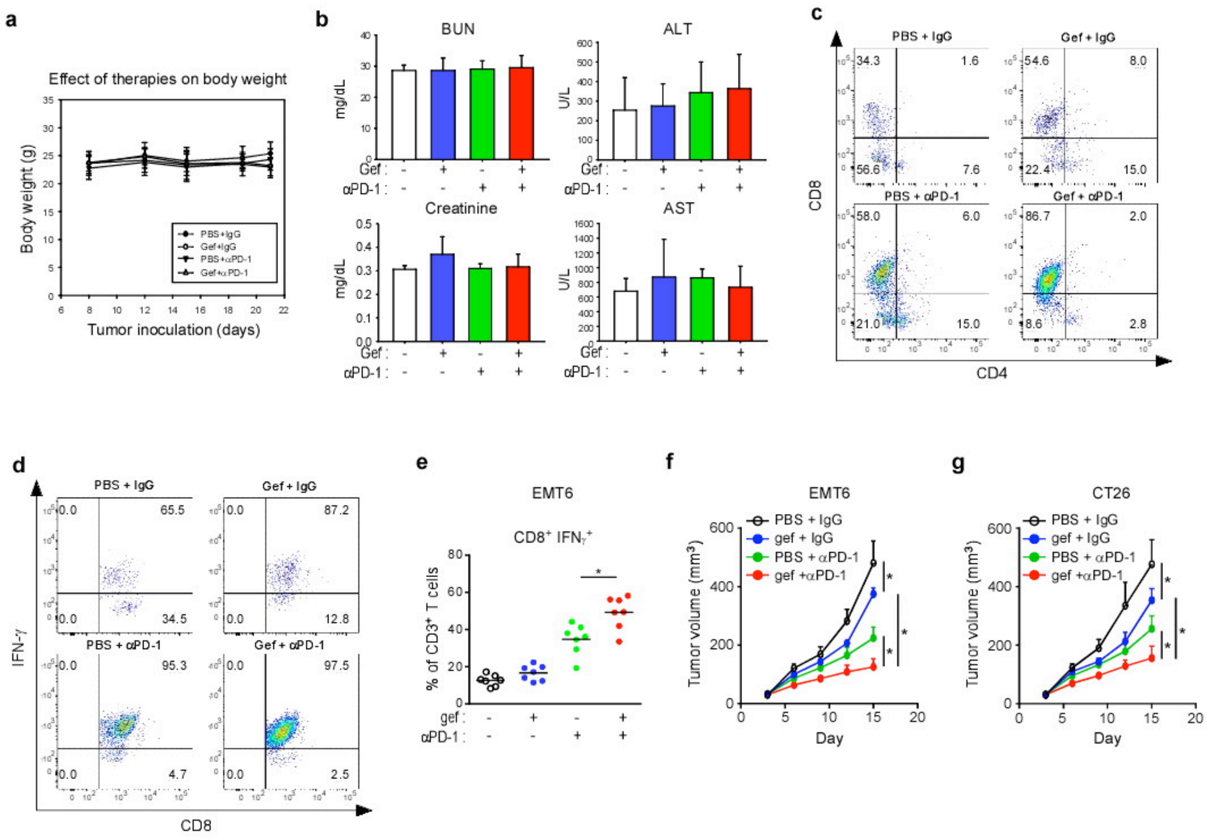

Supplementary Figure 9. T cell profile analysis from tumor infiltrated lymphocytes. (a) The effect of treatment on mice body weight. **(b)** Mice liver and kidney functions were measured at the end of the experiments. **(c)** Flow cytometry of CD8 and CD4 markers on $CD3^+$ T cells isolated from tumors of gefitinib and/or anti-PD-1 antibody treated mice. **(d)** Flow cytometry of IFNγ and CD8 markers on $CD8^+$ $CD4^-$ T cells isolated from tumors of gefitinib and/or anti-PD-1 antibody-treated mice. Error bars represent mean ±SD of 3 independent experiments. **(e)** Intracellular cytokine stain of $IFN\gamma^+$ $CD8^+$ in $CD3^+$ T cell populations from the isolated TIL. **(f)** The tumor growth of EMT6 cells in gefitinib and/or anti-PD-1 antibody treated BALB/c mice. **(g)** The tumor growth of CT26 cells in gefitinib and/or anti-PD-1 antibody treated BALB/c mice.

Fig.1a

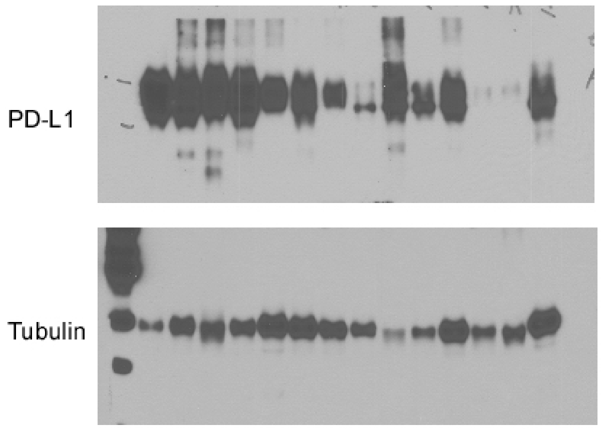

Fig.1h

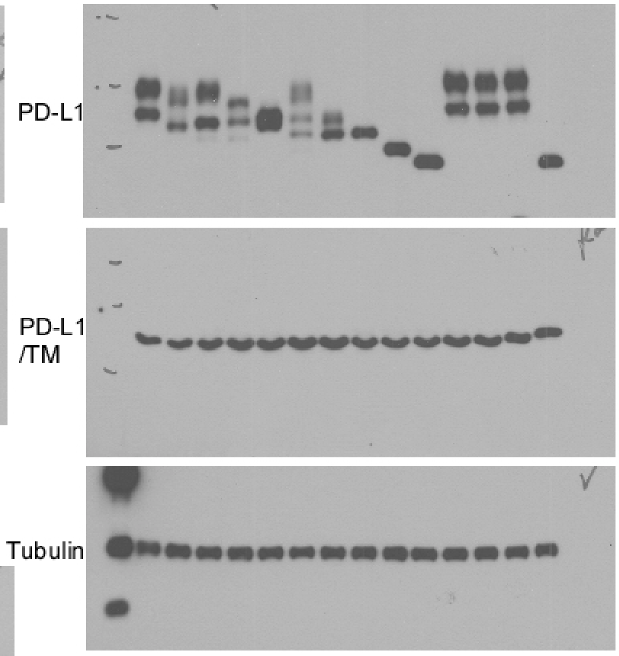

Fig.1b

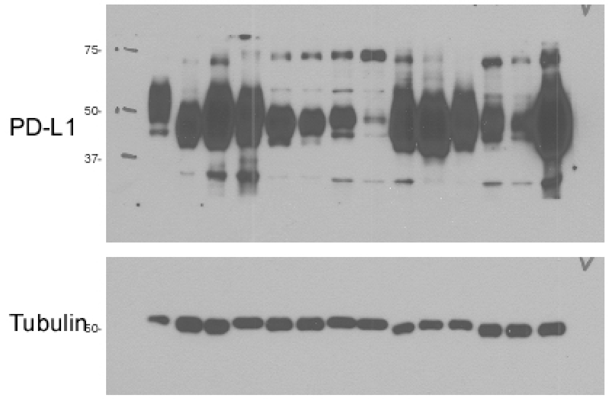

Fig.1e

07-13-2014

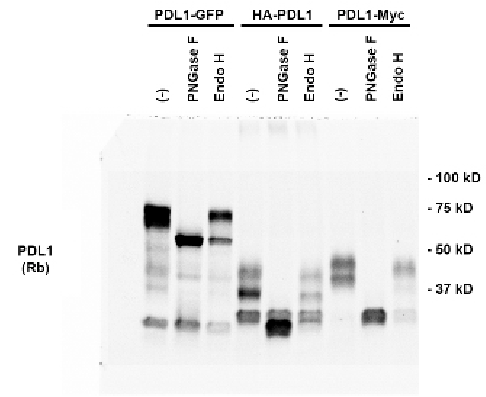

Fig.1c

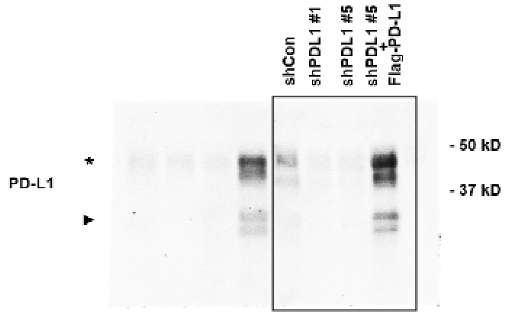

Fig. 2a

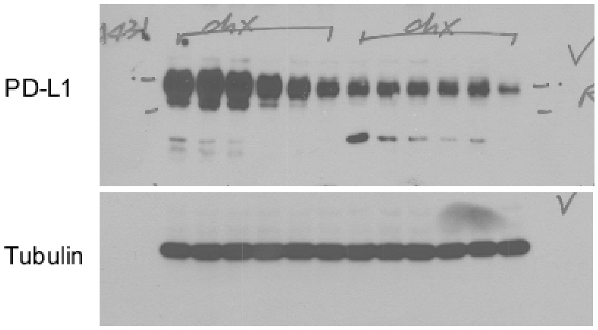

Fig. 2b

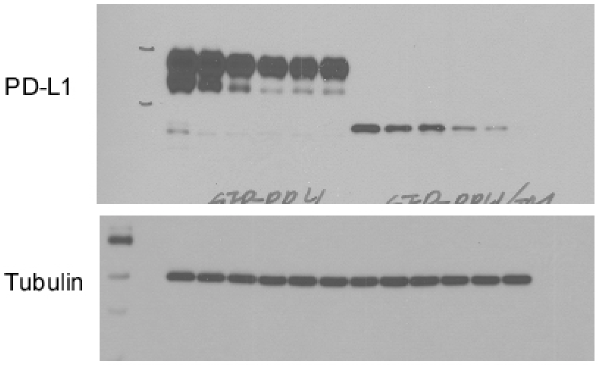

Fig. 2g

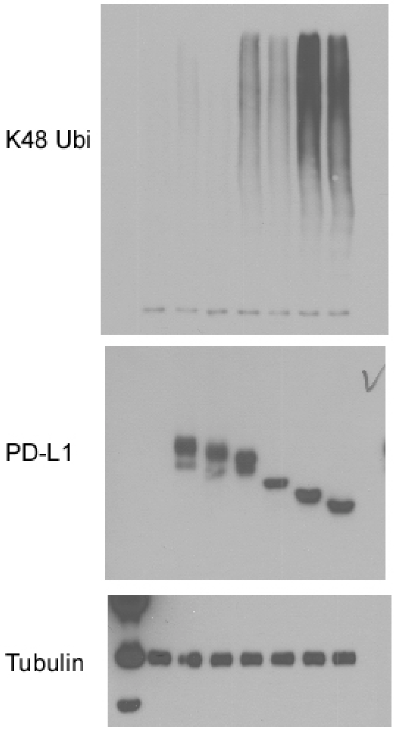

Fig. 2c

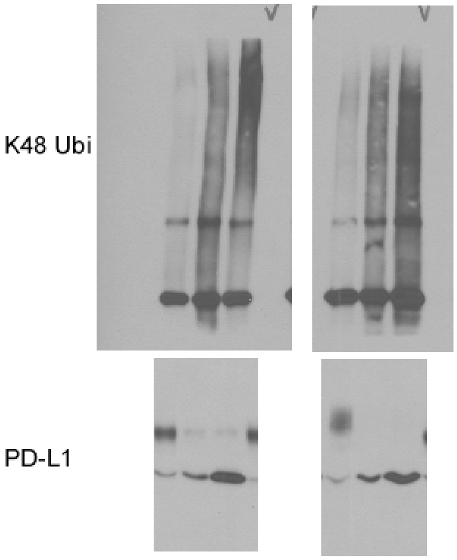

Fig. 2e

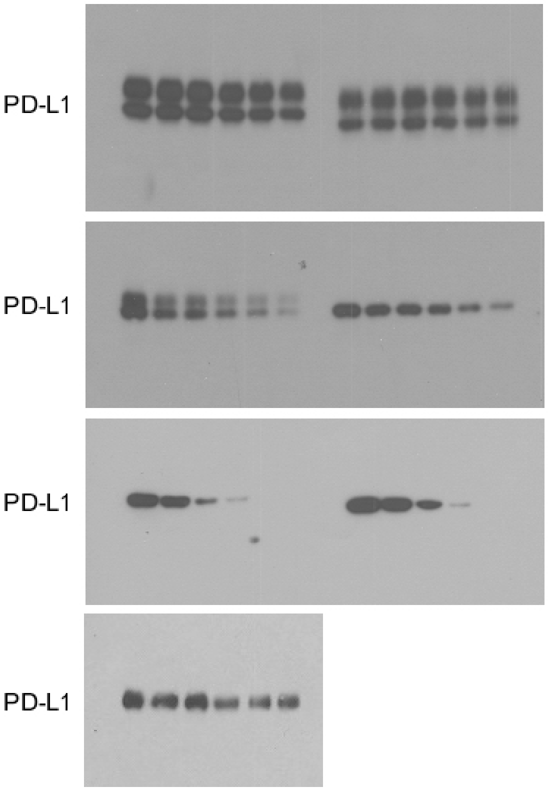

Fig. 3c

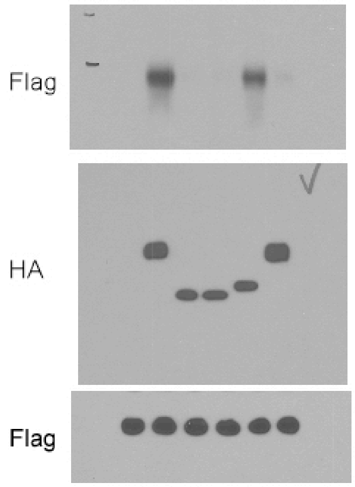

Fig. 3d

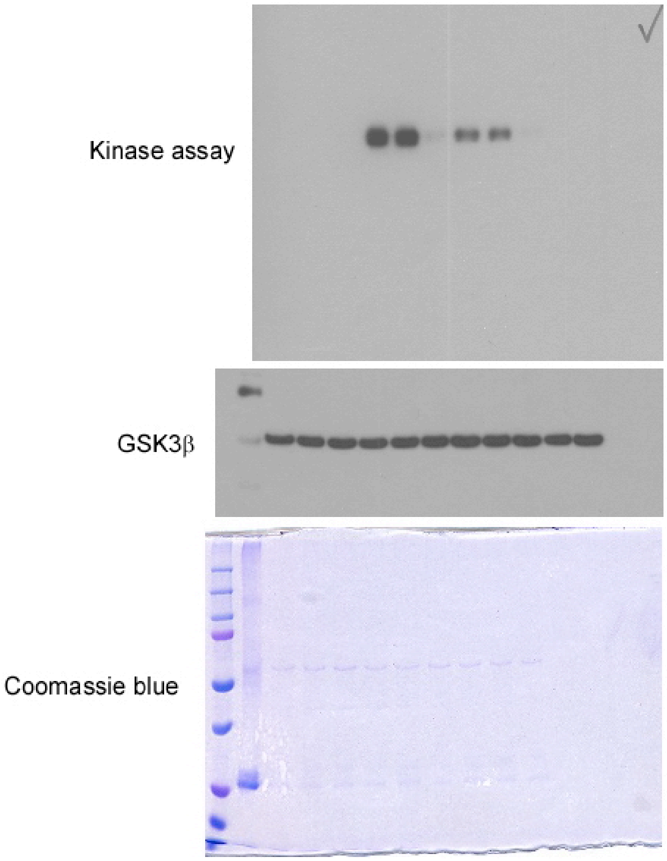

Fig. 3e

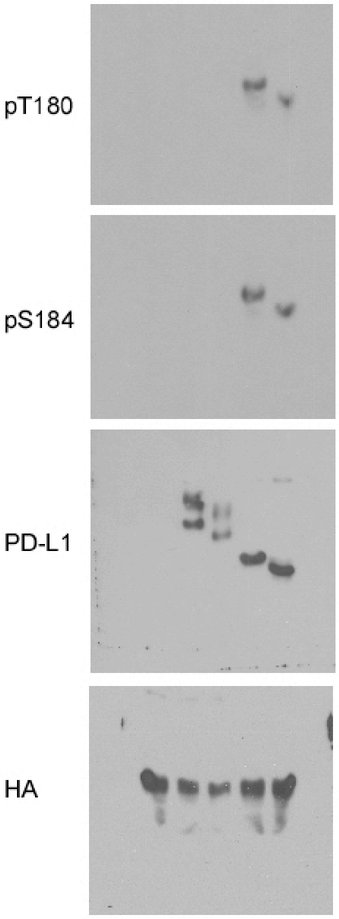

Fig. 4a

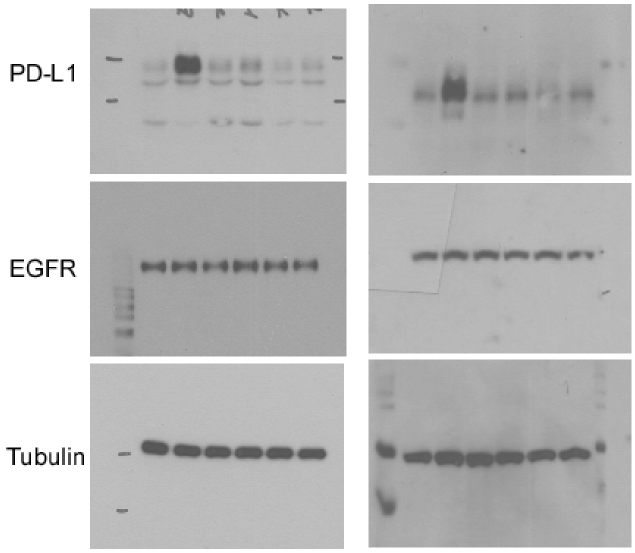

Fig. 4b

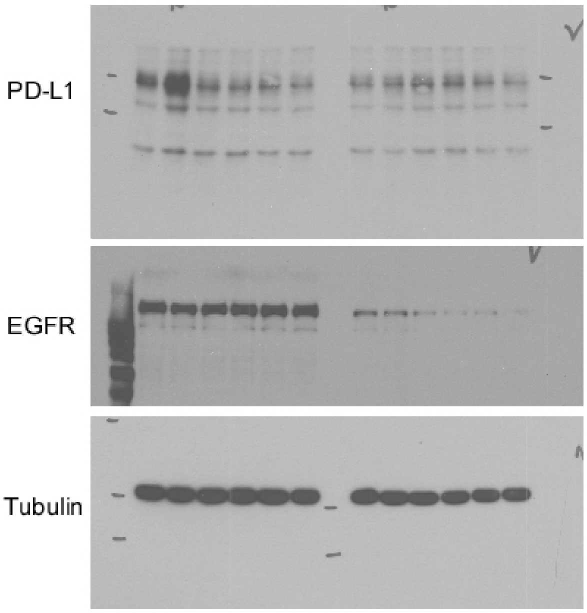

Fig. 4e

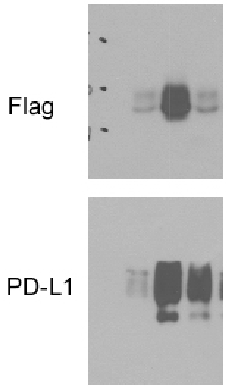

Fig. 4d

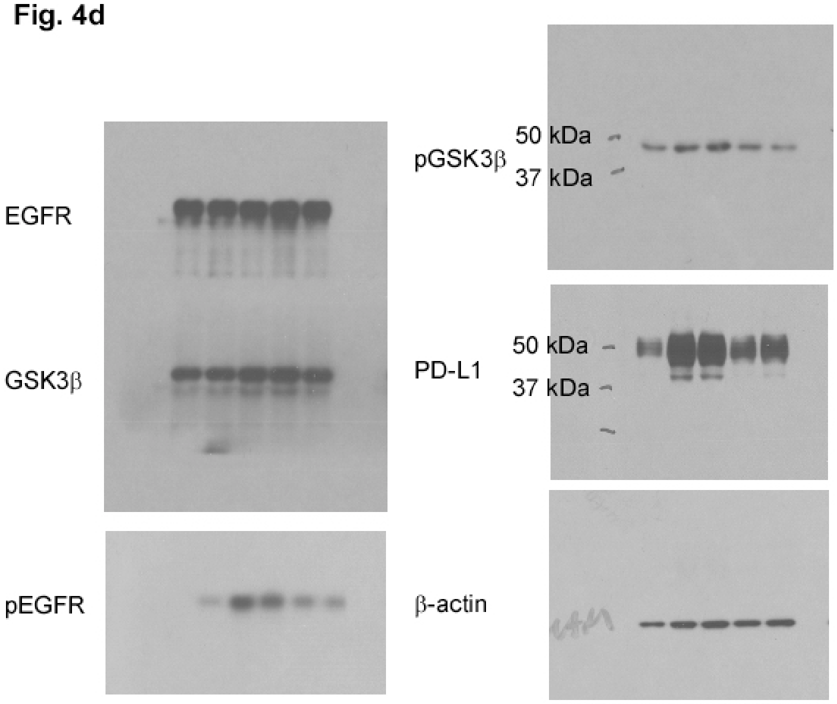

Fig. 5a

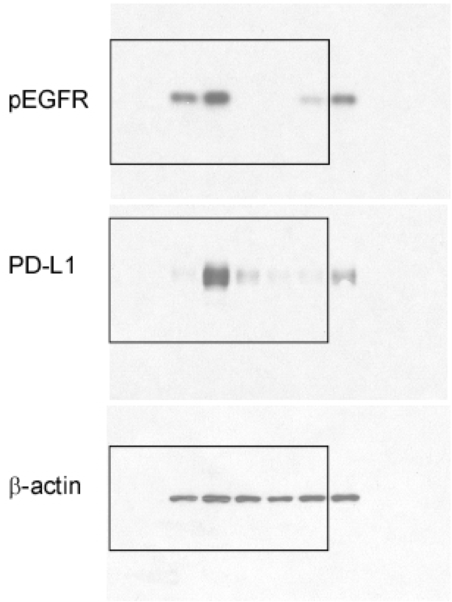

Fig. S1a

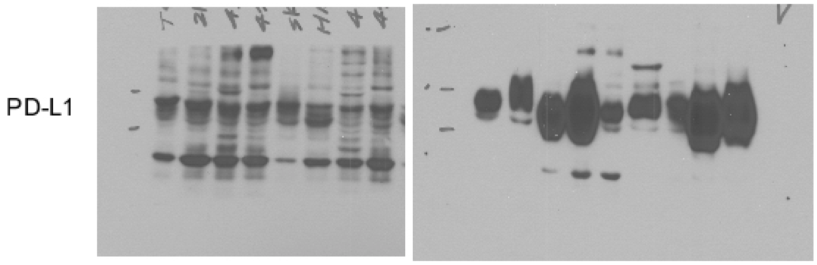

Fig. S1b

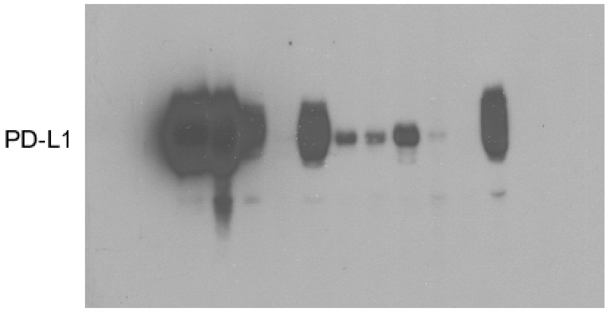

Fig. S2a

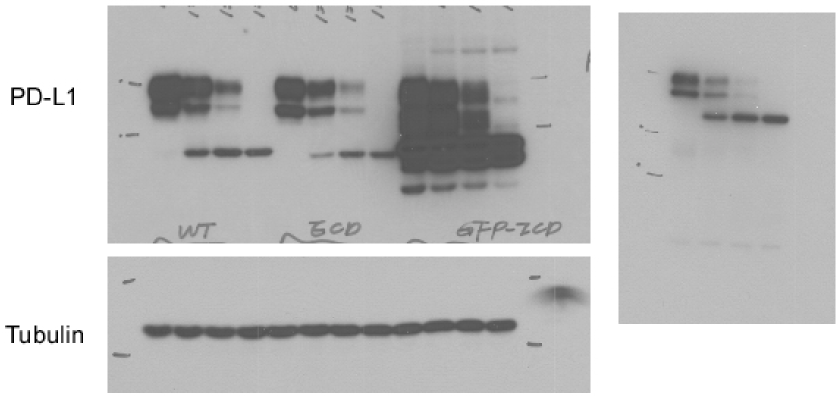

Fig. S2b

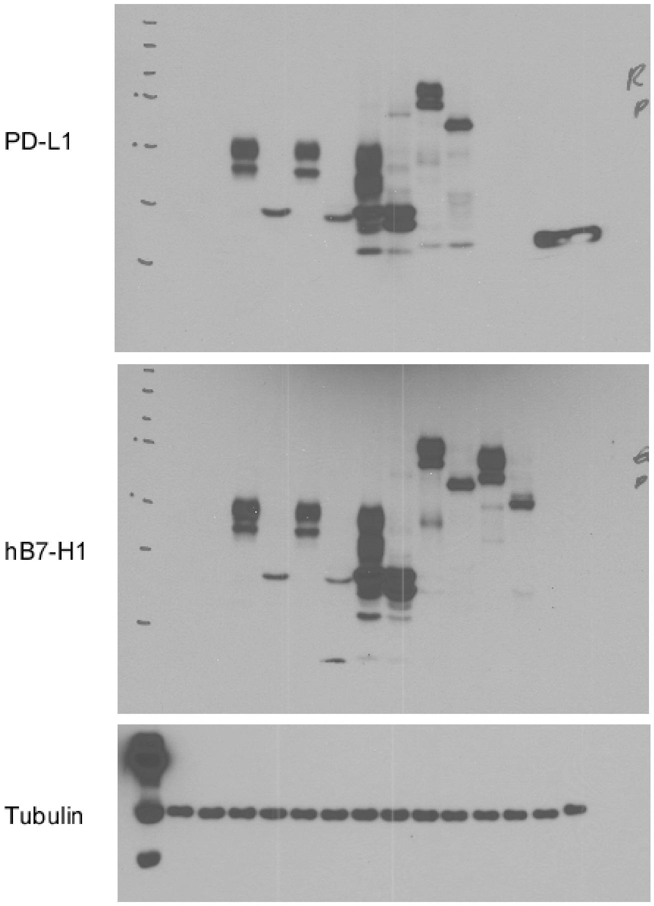

Fig. S5a

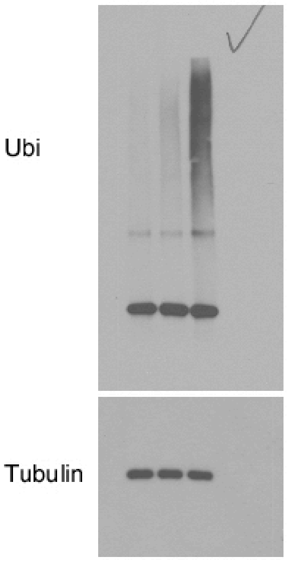

Fig. S5c

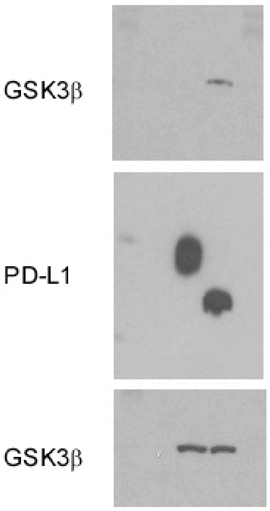

Fig. S5f

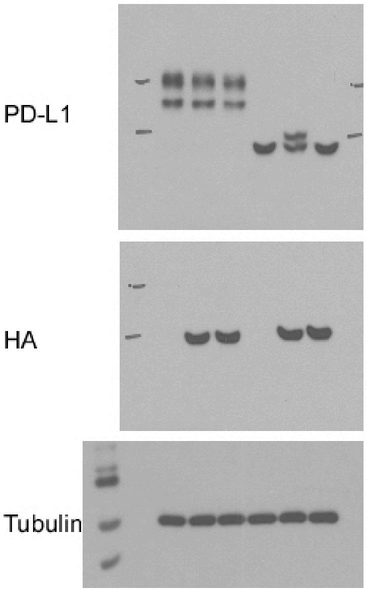

Fig. S5g

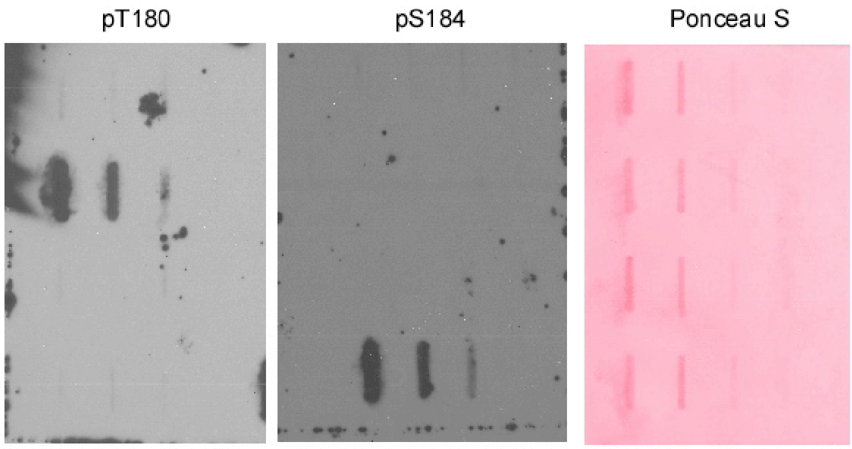

Fig. S6a

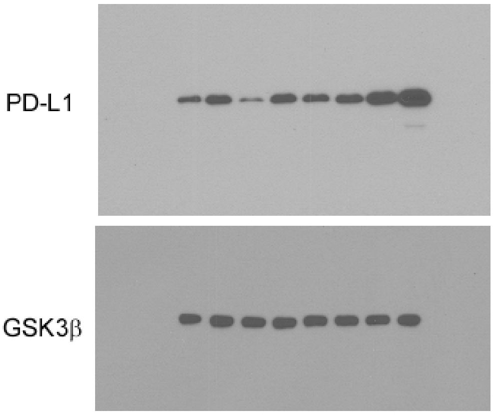

Fig. S6c

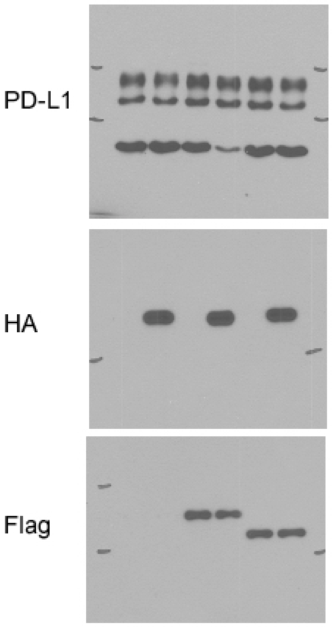

Fig. S6d

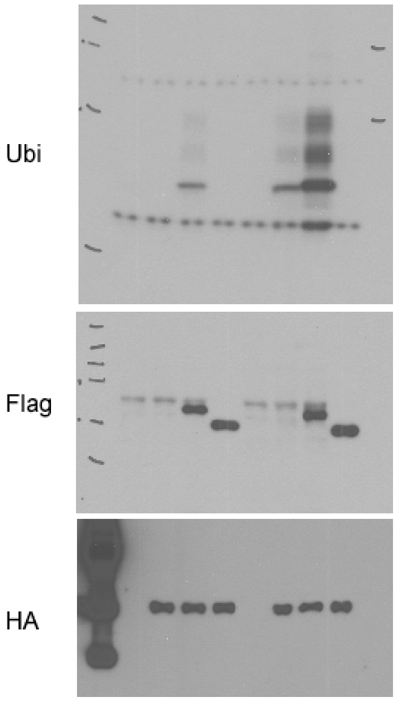

Fig. S6e

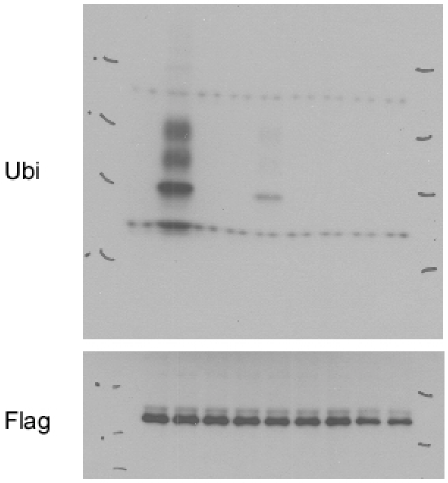

Fig. S7g

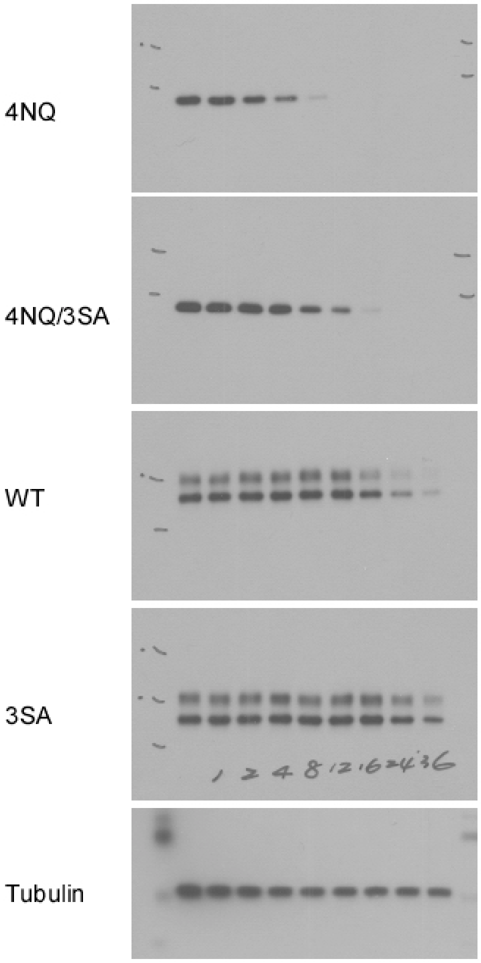

Fig. S7h

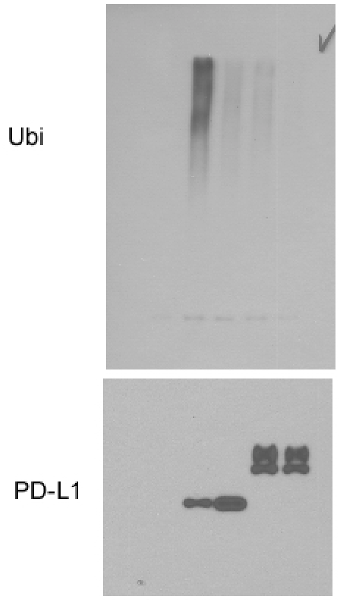

Fig. S8a

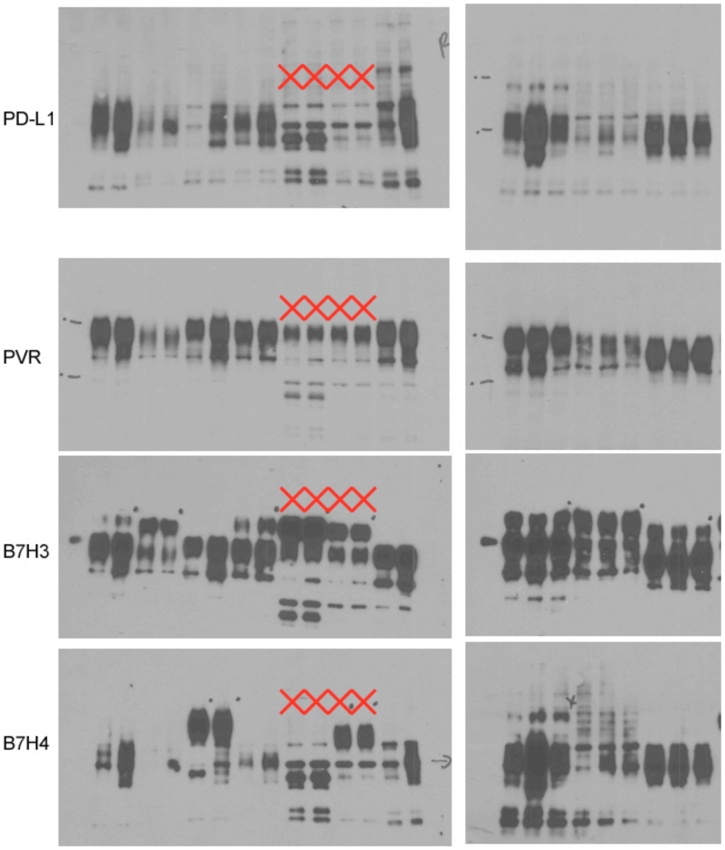

Fig. S8b

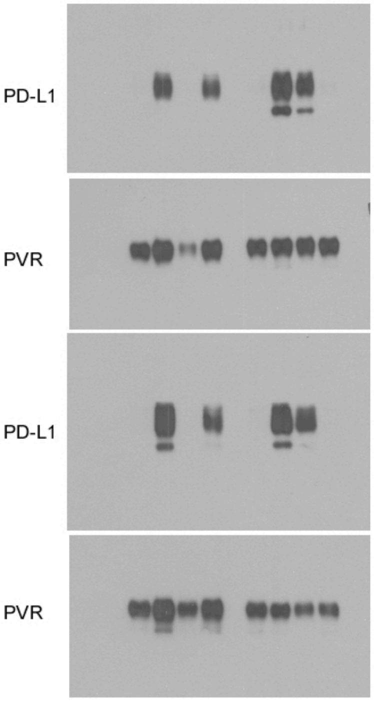

Supplementary Figure 10. Uncropped scans of the Western blots shown in the indicated figures.

Supplementary Table 1. Correlations between expression levels of PD-L1, p-EGFR (Tyr 1068), p-GSK3β (Ser 9), and granzyme B expression in surgical specimens of breast cancer.

		No. expression of PD-L1 (%)			
		− / +	++ /+++	Total	*P* value
p-EGFR	−/ +	26 (45.6%)	28 (25.2%)	54 (32.1%)	*P* = 0.007
	++ /+++	31 (54.4%)	83 (74.8%)	114 (67.9%)	
	Total	57 (100%)	111 (100%)	168 (100%)	
p-GSK3β	− / +	27 (42.9%)	4 (5.2%)	31 (22.1%)	*P* = 0.0001
	++ /+++	36 (57.1%)	73 (94.8%)	109 (77.9%)	
	Total	63 (100%)	77 (100%)	140 (100%)	
Granzyme B	−/ +	50 (68.5%)	92 (81.4%)	142 (76.3%)	*P* = 0.043*
	++ /+++	23 (31.5%)	21 (18.6%)	44 (23.7%)	
	Total	73 (100%)	113 (100%)	186 (100%)	

P, Pearson Chi-Square test; *inverse correlation between PD-L1 and granzyme B. –/+, negative or low expression; ++/+++, medium or high expression.
